# Supplementary material for: From sequence to enzyme mechanism using multi-label machine learning
Source: BMC Bioinformatics. 2014 May 19;15:150. doi: 10.1186/1471-2105-15-150 (PMC4229970; doi:10.1186/1471-2105-15-150)
Supplement: Additional file 2 — Java code of ml2db. Additional file ml2db_code.tar.gz contains the Java source code to run the multi-label machine learning experiments and save the results to database. The code’s Javadoc is included. [file 1471-2105-15-150-S2.zip › additional file 2/ml2db/ml2db/doc/main/ml2db/al/utility/hybrid/GlobalDistanceWithConfidenceMetric.html]

GlobalDistanceWithConfidenceMetric


JavaScript is disabled on your browser.


- Overview
- Package
- Class
- Use
- Tree
- Deprecated
- Index
- Help

- Prev Class
- Next Class

- Frames
- No Frames

- All Classes

- Summary:
- Nested |
- Field |
- Constr |
- Method

- Detail:
- Field |
- Constr |
- Method


main.ml2db.al.utility.hybrid

## Class GlobalDistanceWithConfidenceMetric

- java.lang.Object
- - main.ml2db.al.utility.UtilityMetric
  - - main.ml2db.al.utility.globaldistance.GlobalDistanceMetric
    - - main.ml2db.al.utility.hybrid.GlobalDistanceWithConfidenceMetric

- ---

    

  ```
  public class GlobalDistanceWithConfidenceMetric
  extends GlobalDistanceMetric
  ```

  Combines a Positioning metric (a metric that considers positively if an
  instance is close to other unlabelled instances and distant from other
  labelled instances) with the average certainty of the instance prediction
  (that is, how distant the average of confidence over all labels is from 50%)

  Version:
  :   7 Jul 2011

  Author:
  :   Luna De Ferrari ldf(at)st-andrews.ac.uk

- - ### Field Summary

    Fields

    | Modifier and Type | Field and Description |
    | --- | --- |
    | `static java.lang.String` | `NAME` |

    - ### Fields inherited from class main.ml2db.al.utility.globaldistance.GlobalDistanceMetric

      `STANDARD_BALANCE`
  - ### Constructor Summary

    Constructors

    Constructor and Description

    `GlobalDistanceWithConfidenceMetric()`
  - ### Method Summary

    Methods

    | Modifier and Type | Method and Description |
    | --- | --- |
    | `ConfidenceCalculator` | `getConfidenceCalculator()` |
    | `java.lang.String` | `getMetricName()` |
    | `double` | `getUtility(mulan.classifier.MultiLabelLearner trainedLearner, IdInstance instance)` Gets a measure of how distant the instance is from other unlabelled instances (good if it's close to them) and labelled instances (bad if it's close to them) and multiplies it by the average confidence certainty (see Confidence class). |
    | `void` | `refresh(IdMultiLabelInstances labelled, IdMultiLabelInstances unlabelled, IdInstance instanceLabelled)` Refresh the set of labelled and unlabelled instances |

    - ### Methods inherited from class main.ml2db.al.utility.globaldistance.GlobalDistanceMetric

      `getBalance, getDistanceCalculator, getDistances, getLabelled, getLabelledDistances, getPositionsStore, getUnlabelledDistances, initialise, initialise, isEmptyLabelledSet, main, serializeUnlabelledPositions, setDebug, setEmptyLabelledSet, setInstances, updateDistances`
    - ### Methods inherited from class main.ml2db.al.utility.UtilityMetric

      `getUnlabelled, setUnlabelled`
    - ### Methods inherited from class java.lang.Object

      `equals, getClass, hashCode, notify, notifyAll, toString, wait, wait, wait`

- - ### Field Detail


    - #### NAME

      ```
      public static final java.lang.String NAME
      ```

      See Also:
      :   Constant Field Values
  - ### Constructor Detail


    - #### GlobalDistanceWithConfidenceMetric

      ```
      public GlobalDistanceWithConfidenceMetric()
      ```
  - ### Method Detail


    - #### getConfidenceCalculator

      ```
      public ConfidenceCalculator getConfidenceCalculator()
      ```


    - #### getMetricName

      ```
      public java.lang.String getMetricName()
      ```

      **Overrides:**
      :   `getMetricName` in class `GlobalDistanceMetric`


    - #### getUtility

      ```
      public double getUtility(mulan.classifier.MultiLabelLearner trainedLearner,
                      IdInstance instance)
      ```

      Gets a measure of how distant the instance is from other unlabelled
      instances (good if it's close to them) and labelled instances (bad if
      it's close to them) and multiplies it by the average confidence certainty
      (see Confidence class).

      **Overrides:**
      :   `getUtility` in class `GlobalDistanceMetric`

      Parameters:
      :   `balance` - if > 0.5, the distance from labelled instances counts more
          than the closeness to many unlabelled instances. The other way
          round if < 0.5.
      :   `distancesFromLabelled` - the distances between this instance and each labelled instance
      :   `distancesFromUnlabelled` - the distances between this instance and each unlabelled
          instance

      Returns:
      :   a value representing the position of this instance in relation to
          other labelled and unlabelled instances.


    - #### refresh

      ```
      public void refresh(IdMultiLabelInstances labelled,
                 IdMultiLabelInstances unlabelled,
                 IdInstance instanceLabelled)
      ```

      **Description copied from class: `UtilityMetric`**

      Refresh the set of labelled and unlabelled instances

      **Overrides:**
      :   `refresh` in class `GlobalDistanceMetric`


- Overview
- Package
- Class
- Use
- Tree
- Deprecated
- Index
- Help

- Prev Class
- Next Class

- Frames
- No Frames

- All Classes

- Summary:
- Nested |
- Field |
- Constr |
- Method

- Detail:
- Field |
- Constr |
- Method
